# Efficacy of perioperative esketamine on postoperative depression: a systematic review and meta-analysis

**DOI:** 10.3389/fpsyt.2025.1476449

**Published:** 2025-04-15

**Authors:** Hao-Yan Li, Wen-Jing Xu, Ya-Mei Wang, Shuang Xie, Huan-Liang Wang

**Affiliations:** Department of Anesthesiology, The Second Affiliated Hospital of Hainan Medical University, Haikou, Hainan, China

**Keywords:** esketamine, postoperative, depression, anesthesia, meta-analysis

## Abstract

**Background:**

Postoperative depression (POD) represents a serious complication in surgical patients, exacerbating morbidity and mortality rates while imposing a substantial economic burden on healthcare systems. Despite its widespread clinical use, the role of esketamine, an NMDA receptor antagonist with rapid antidepressant effects, remains understudied in perioperative settings. Therefore, we conducted a systematic review and meta-analysis to assess the efficacy of esketamine on postoperative depression. To evaluate the effect of esketamine on the incidence and severity of postoperative depression in different types of surgery by randomized controlled trial, investigate whether esketamine can effectively reduce the postoperative depression score and the incidence of postoperative depression in the short and long term after use, to promote the application of perioperative analgesia-antidepressant combination.

**Method:**

Searched PubMed, the Cochrane Library, the Web of Science, and Medline to identify randomized controlled trials using the drug of esketamine and analyzed the data using Review Manager 5.3.

**Results:**

We included a total of 8 randomized controlled trials involving 1724 patients who met the criteria. The meta-analysis revealed that esketamine treatment, compared with control groups, significantly reduced POD. Improvements were observed at 1 week (RD -0.09, 95% CI [-0.13, -0.05], P < 0.0001, I²=84%), 2 weeks (RD -0.08, 95% CI [-0.13, -0.03], P < 0.00001, I²=97%), and long-term follow-up (RD -0.06, 95% CI [-0.10, -0.02], P=0.0002, I²=79%).

**Conclusion:**

Esketamine demonstrates efficacy in reducing POD incidence and severity, although its use is associated with an increased risk of adverse effects. Also, the method of drug injection, the duration of administration and the number of doses may have an effect on the results. Therefore, further exploration of appropriate dosing regimens and multi-modal strategies is necessary to mitigate adverse effects.

**Systematic review registration:**

https://www.crd.york.ac.uk/PROSPERO/, identifier CRD42024506329.

## 1 Introduction

Surgical interventions have become a cornerstone of modern medicine, anesthesia allowed them to perform more complex, invasive, and precise maneuvers than they had dared to attempt before (1). Perioperative treatment is a great challenge for anesthesiologists because it causes psychological stress reactions such as anxiety and depression and may increase the risk of postoperative complications (2).

Depression is a heterogeneous disorder that can manifest as low mood, loss of interest and pleasure in normal and pleasant activities, difficulty in thinking and decision-making, and disturbances in appetite and sleep (3, 4). Postoperative depression (POD) is defined as the onset or exacerbation of depressive symptoms within 30 days following surgery, due to its transient nature and direct association with surgical stress, anesthesia, and postoperative recovery, remains a serious problem for surgical patients. POD is associated with impaired immune function, elevated infection risks, and worsened oncologic outcomes (5). Major depressive disorder (MDD) is a common POD manifestation and can further aggravate its morbidity and mortality. MDD is one of the chronic mental disorders that induce disability, and projected to become the leading cause of global disability by 2030 (6), affecting 15-20% of the population and accounting for 50% of psychiatric suicides (7–12)”.

Despite the administration of diverse monoaminergic antidepressants, approximately one-third of MDD patients fail to achieve symptom relief. Patients with a major depressive episode who do not respond to an adequate duration of antidepressant treatment involving two medications are considered to have developed "treatment-resistant depression" (TRD) (13). Therefore, how to effectively prevent and lower the risk of postoperative depression is particularly important. Choosing appropriate anesthetic drugs during the perioperative period to prevent the occurrence of postoperative depression in patients has become an issue that anesthesiologists should consider.

Esketamine, the S-enantiomer of ketamine, exerts rapid antidepressant effects via N-methyl-D-aspartate (NMDA) receptor antagonism, enhancing prefrontal glutamate release and synaptic plasticity through BDNF-mTOR pathways (14–16). A study on the variability of patient responses to esketamine treatment using a machine learning model further suggest its potential for personalized psychiatry (17), while its dissociation and agitation are not very common among the side effects of esketamine, and there is no potential for addiction after its use. Therefore, these pieces of evidence indicate that esketamine for the treatment of depression is mild and acceptable (18).

Given the previously reported evidence regarding these complementary effects of esketamine as a postoperative antidepressant, intraoperative combination use of esketamine may be a promising approach to reduce the risk of postoperative major depression. However, there is a lack of meta-analyses on the efficacy of esketamine in the treatment of postoperative major depression. Therefore, the purpose of this study was to conduct a systematic review and meta-analysis of randomized controlled trials (RCTS) to investigate the efficacy of esketamine on postoperative depression, as well as the effects of different doses of esketamine on postoperative depression in patients and the risk of adverse effects.

## 2 Method and analysis

This meta-analysis was performed according to the recommendations in the Preferred Reporting Items for Systematic Reviews and Meta Analyses (PRISMA) statement and the guidelines described in the Cochrane Handbook (19).

### 2.1 Patient and public involvement

This work was based on published research data. Therefore, no patient or public involvement is required. Relevant results will be published in peer-reviewed journals.

### 2.2 Search strategy

We limited the search to peer-reviewed articles that were clinical studies with the use of a randomized controlled trial design and were published in English in the Cochrane Central Register of Controlled Trials, including four English electronic databases (PubMed, Cochrane Library, web of science, Medline). The search strategy was performed specifically for each database and included a combination of the medical subject headings and free text terms for (“esketamine” OR “s-ketamine” OR “L-ketamine” OR “ (–)-ketamine”) and (“depression” OR”depressive” OR “depressed “OR “postoperative depression”). The deadline for all retrieval was December 2023.

### 2.3 Inclusion/exclusion criteria

The following criteria were included:

(1) intervention: esketamine; (2) comparison: placebo, no intervention or other sedative hypnotics; (3) type of study: randomized controlled trial (RCT); (4) Comparison of the effects of postoperative depression with esketamine (experimental group) and the control group (no intervention or other sedative-hypnotic agents). (5) Postoperative depression (POD) diagnosed using validated scales (e.g., HAMD-17, EPDS) within 30 days postoperatively.

Exclusion criteria were as follows:

(1) Article did not conduct a randomized controlled trial; (2) Experimental design cannot extract relevant data on the results; (3) The article was themed in the form of a review or case report. (4) Specific types of surgeries or comorbidities may affect generalizability. (5) Patients with pre-existing psychiatric disorders.

### 2.4 Data extraction

We classified the extracted data using Microsoft Excel and then transcribed the data into the Review Manager (version 5.3) for statistical analysis. The data analyzed included name of the author, number and genders of the patients, types of surgery, types of anesthesia, time and doses of intervention for the esketamine group and the control group, postoperative depression scores, pain intensity scores, postoperative adverse effects, and time for follow-up. Two independent reviewers screened all the titles and abstracts to determine potential eligible articles. They independently applied the eligibility criteria to perform the final selection. When discrepancies occurred between both reviewers regarding the inclusion of the articles, they discussed and identified the reasons to either include or exclude the articles and then made the final decision. If they could not reach an agreement, the final decision was based on a third reviewer.

All rating scales on changes in depressive mood were extracted from the selected studies, including Edinburgh Postpartum Depression Scale (EPDS) scores, Montgomery-Absberg Depression Scale (MADRS), Beck Depression Scale (BDI), and Hamilton Depression Scale (HAMD-17) scores. Secondary outcomes included postoperative pain (visual analog scale) and adverse effects (e.g., headache, nausea). Three treatment time points (within 1 week, 2 weeks, and over 4 weeks) were selected to evaluate the effect of esketamine.

### 2.5 Strategy for data synthesis

The data were analyzed using the random-effects model to determine the pooled risk ratio (RR), mean difference (MD), and standardized mean difference (SMD). Additionally, the 95% confidence interval (CI) was reported for each outcome. A random-effects model was selected due to anticipated clinical heterogeneity (e.g., varied surgical types and dosing regimens).

We were unable to perform subgroup analyses of the effects of esketamine on depression because there were only one or two studies in each surgical procedure.

To further investigate the safety of perioperative use of esketamine for antidepressant effects, we conducted a subgroup analysis of adverse reactions following esketamine use. Statistical analysis was performed using the Review Manager (RevMan). Statistical significance was set with the probability value (p) of less than 0.05.

### 2.6 Risk of bias in individual studies

**Figure 1:**
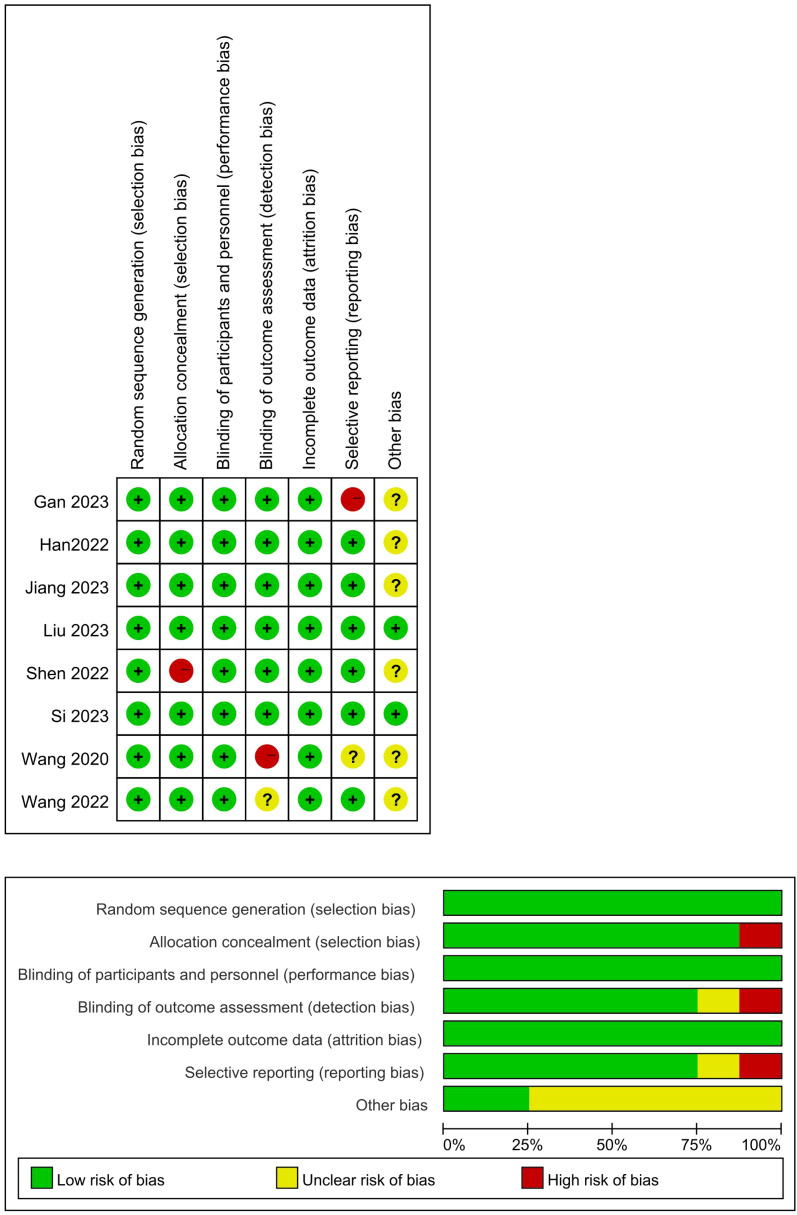
Quality assessment of the included studies. (+: Low risk of bias; –: High risk of bias; Yellow grid: Unclear risk of bias).

We assessed the risk of bias within individual trials using the Cochrane risk of bias tool for randomized controlled trials and independent assessment was conducted by two trained reviewers. Specifically, the risk of bias for each trial was classified as “low”, “high”, and “some concerns”. The risk of bias tool assesses indicators of selection bias, experimental bias, detection bias, and reporting bias. Any disagreement was resolved through discussion to reach a consensus (**Figure 1**).

### 2.7 Assessment of heterogeneity

We assessed between-study heterogeneity using the I^2^ statistic, with 50% or higher values indicating significant heterogeneity.

## 3 Results

### 3.1 Study search and characteristics

**Figure 2:**
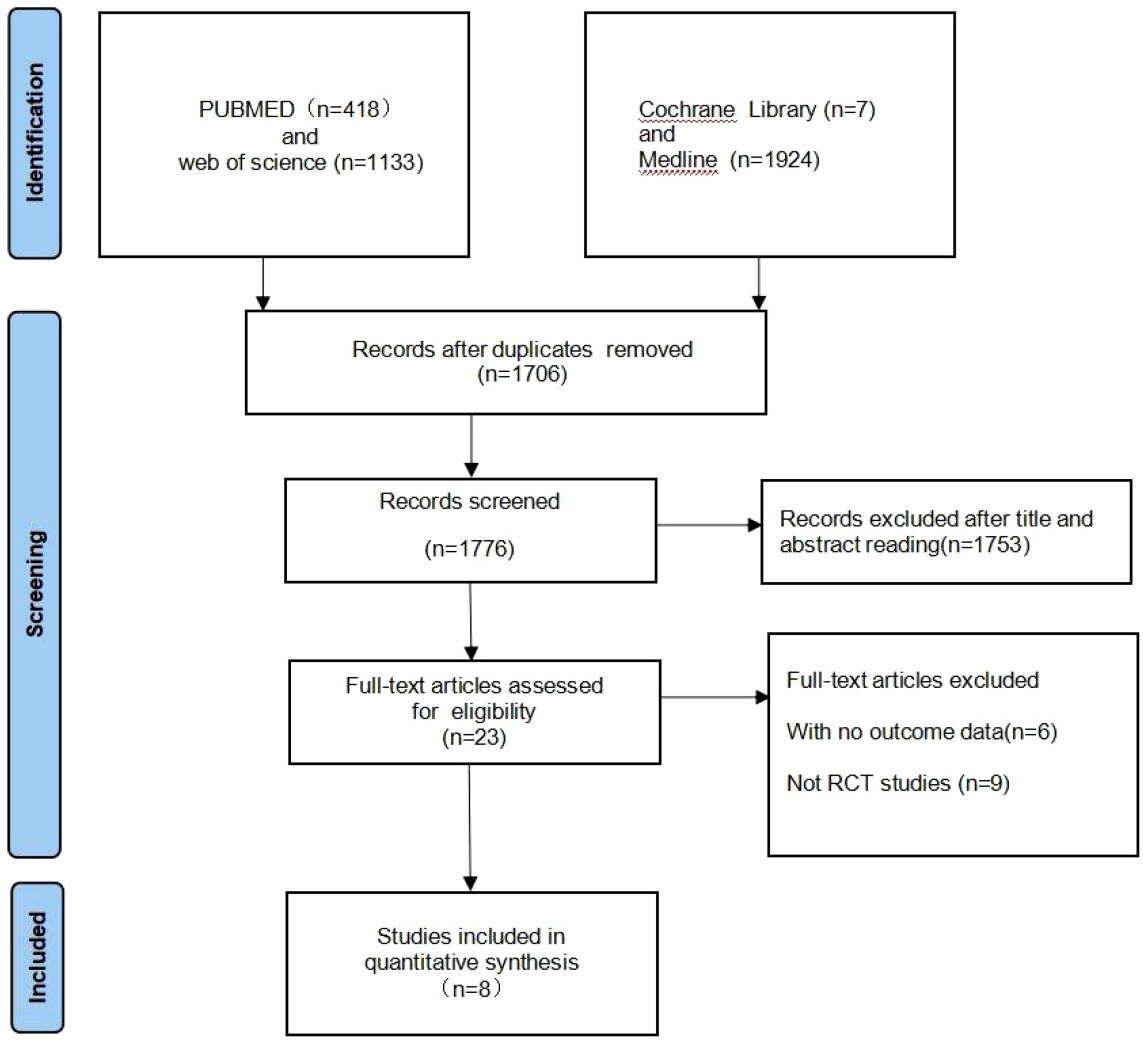
Flow chart for study selection. Total number of studies identified, screened, deemed eligible, and ultimately included is summarized.

A total of 3482 records were identified for preliminary screening. After screening the titles and abstracts, 8 eligible studies were included in this meta-analysis (**Figure 2**). Finally, the current meta-analysis included a total of 8 RCTs published between 2020 and 2023 with 1,724 participants (**Table 1**) (20–27).

**Table 1:** Characteristics of included studies.

First author	Year	Country	Study Design and sample size (S/C)	Age (S/C)	Sex	Surgery	Anesthesia	Ways of Esketamine injection	Time point of intervention	Intervention (S vs. C)	Depression Measurement	Pain Measurement	Follow up Period
Han (20)	2022	china	RCT 275(122/153)	31.64 ± 3.93	F	cesarean section	Spinal anesthesia	PCIA	after suturing the skin incision	S-ketamine 0.5mg/kg + sufentanil 2μg/kg + tropisetron 10mg vs. sufentanil 2μg/kg + tropisetron 10mg	EPDS	VAS	Post-op days 3,14 and 28
				31.85 ± 4.16									
Wang (21)	2022	china	RCT 156(39/40/38/39)	27.9 ± 6.1	F	cesarean section	Spinal anesthesia	PCIA	at the time of surgical suture	S-ket-low(0.1mg/kg)vs. S-ket-middle(0.2mg/kg)vs. S-ket-High(0.4mg/kg)vs. sufentanil 1.5μg/kg + totanisoltron 4mg	PPD	/	Post-op week 1 and 6
				28.3 ± 5.9									
				28.8 ± 6.4									
				29.1 ± 5.5									
Gan (22)	2023	china	RCT 151(75/76)	54 ± 11.7	M+F	thoracoscopic lung cancer surgery	General anesthesia	Single dose + Continuous infusion+ PCIA	Intraoperative-ly	S-KET 0.1mg/kg/h IV vs. normal saline	BDI-II	/	Post-op month 1,3 and hospital discharge
				53.8 ± 12.1									
Yang (23)	2023	china	RCT 195(99/99/97)	31.9	F	cesarean section	Spinal anesthesia	PCIA	after child birth	S-ket-High(sufentanil 2.2μg/kg + esketamine 2.0 mg/kg), S-ket-low(sufentanil 2.2μg/kg+ esketamine 1.0 mg/kg) vs. sufentanil 2.2μg/kg	PDS	/	Post-op day7,42
				31.7									
				32.2									
Jiang (24)	2023	china	RCT 105(35/35/35)	32.77 ± 5.25	F	patients with missed miscarriage	General anesthesia	Single dose	induction	Esketamine 0.3mg/kg + propofol 1.5–2mg/kg vs. Dezocine 0.08 mg/kg + propofol 1.5-2mg/kg vs. propofol 2.5–3mg/kg	EPDS	VSA	Post-op day7,42
				32.29 ± 5.15									
				30.71 ± 4.33									
Shen (25)	2022	china	RCT 202(102/100)	28.9 ± 3.9	F	cesarean section	Spinal anesthesia	Single dose	after child birth	0.25 mg/kg esketamine vs. normal saline	EPDS	NRS	Post-op week 1,2 and4
				29.6 ± 3.9									
Liu (26)	2023	china	RCT 123(62/61)	30.3 ± 4.1	F	cesarean section	Spinal anesthesia	Continuous infusion+ PCIA	After clamping the neonatal umbilical cord	100μg of sufentanil, 1.25 mg/kg of esketamine, and 8 mg of ondansetron vs. 100μg of sufentanil and 8 mg of ondansetron	EPDS	NRS	Post-op day 3,42 and months3,6
				29.8 ± 4.2									
Wang (27)	2020	china	RCT 417(104/104/104/105)	48.53 ± 10.0	F	Laparoscopic Total Hysterectomy	General anesthesia	Single dose	after 1h of analgesia	0.5 mg/kg S-ketamine vs. 0.25 mg/kg S-ketamine vs. 0.5 mg/kg racemic ketamine vs. normal saline	HAMD-17	VAS	Post-op days 1,2,3,5and7
				48.11 ± 10.38									
				47.07 ± 10.08									
				46.27 ± 10.83									

### 3.2 Outcomes

All eight studies had a total of 1,724 participants, with 815 participants in the esketamine group and 909 participants in the placebo group. In the eight randomized controlled trials, esketamine was used for the experimental group. There were five studies on postpartum depression, three articles injected esketamine through PCIA, one article gave a single intravenous dose of esketamine, and one was injected with esketamine by continuous intravenous infusion and postoperative PCIA.

The remaining three studies were on thoracoscopic lung cancer, retained abortion, and laparoscopic total hysterectomy respectively. The experimental groups in all the three studies were injected with a single dose of intravenous esketamine. The ASA body status classification varied from I to III in eight randomized controlled trials, with ASA II being the most common. In eight randomized controlled studies, esketamine at 0.5mg/kg (20), or at low doses (0.1mg/kg), Medium dose (0.2mg/kg), High dose (0.4mg/kg) (21) or at a high dose (2.0 mg/kg), A low dose (1.0 mg/kg) combined with sufentanil at 2.2 μg/kg (23), 0.1mg/kg/h (22), 0.3mg/kg (24), 0.25 mg/kg (25), 0.25 mg/kg (27) with a single dose of esketamine and its control group.

**Figure 3:**
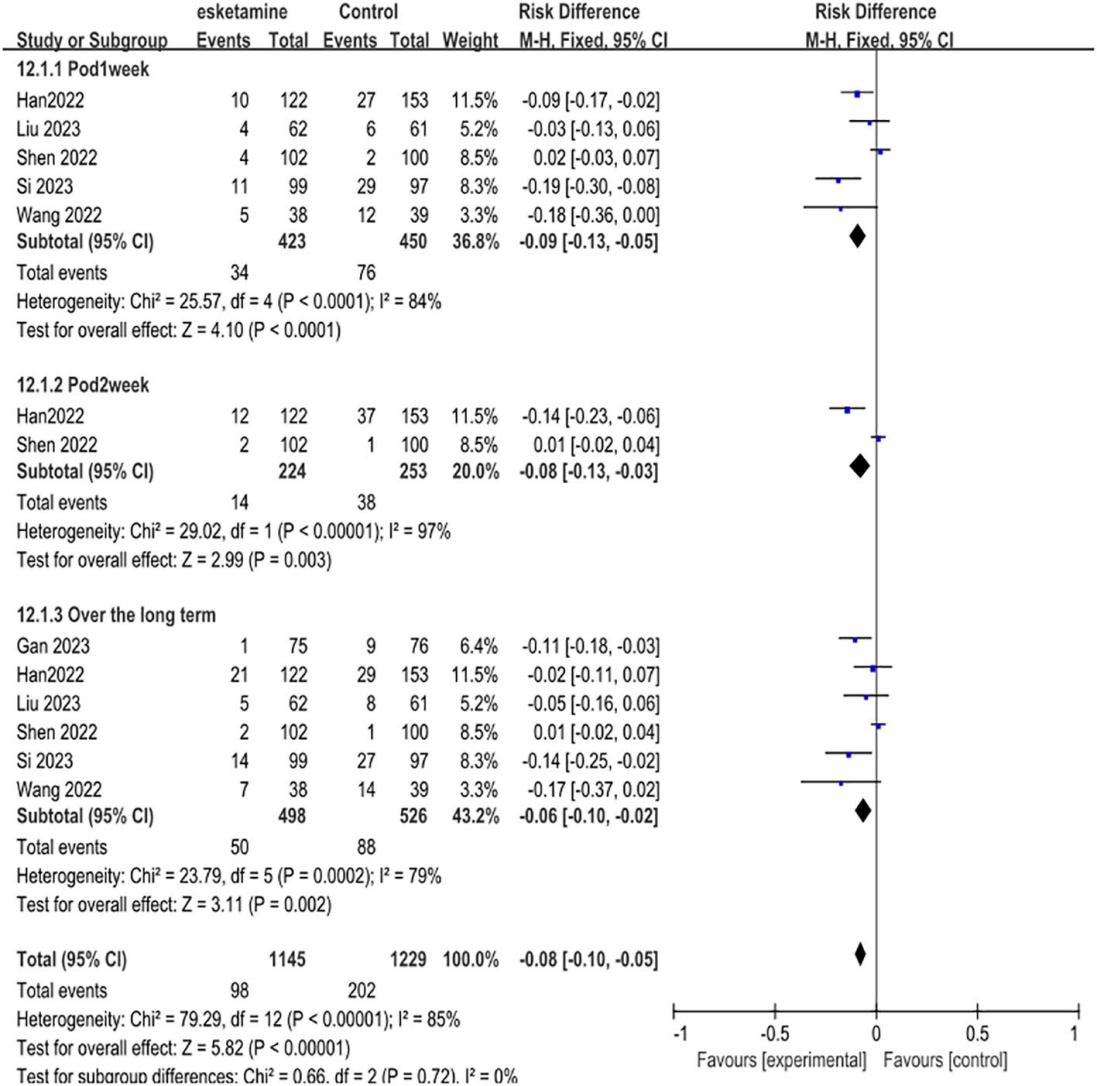
Meta-analysis of postoperative depression occurrence at one week, two weeks, and over the long term.

The first part of our primary meta-analysis investigated the long-term differences in depression scores between esketamine and saline/deszocin/propofol for surgical patients within one week, two weeks, four weeks and beyond respectively. For the high, medium and low doses of esketamine, we included the data of the low dose group in the analysis and used the experimental data of the medium and high dose groups as a reference. From the 8 selected studies, a total of 815 patients received esketamine and 909 patients receiving saline, propofol, or deszocin were treated as control groups. The antidepressant effect was better in the esketamine group compared to the control group. Compared to placebo, POD 1week (RD -0.09, 95% CI [-0.13, -0.05], P <0.0001, I^2^ = 84%), POD2week (RD-0.08,95%CI [-0.13, -0.03], and P <0.00001, I^2^ = 97%) compared to placebo, Over the long term (RD-0.06, 95%CI [-0.10, -0.02], P=0.0002, I^2^ = 79%) had a positive effect (**Figure 3**).

**Figure 4:**
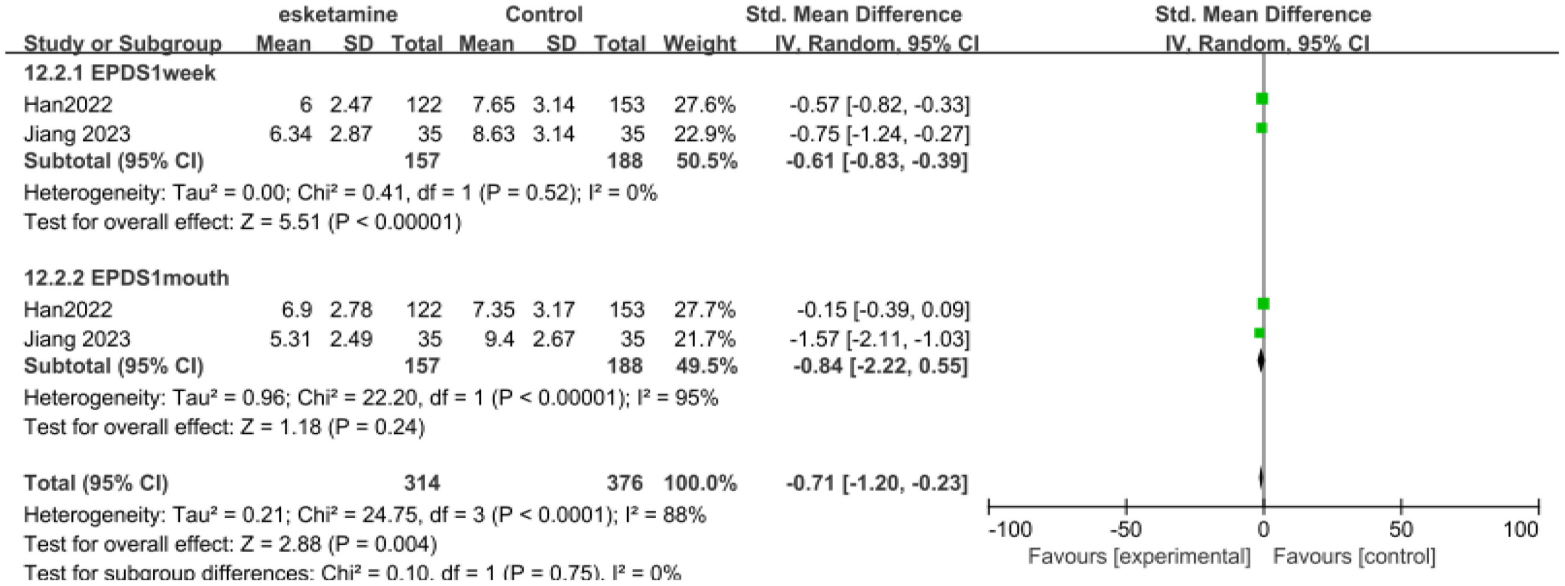
Meta-analysis of EPDS scores at one week and one month of follow-up.

Only two studies had data for the EPDS score, with the duration within one week and one month respectively (**Figure 4**). 157 patients received esketamine and 188 patients receiving saline or propofol were included as controls. On the EPDS, 1week (SMD-0.61, 95%CI [-0.83, -0.39], P=0.52, I^2^ = 0), EPDS 1month (SMD-0.84,95%CI [-0.22,0.55], P <0.00001, I^2^ = 95%).

### 3.3 Adverse effects

**Figure 5:**
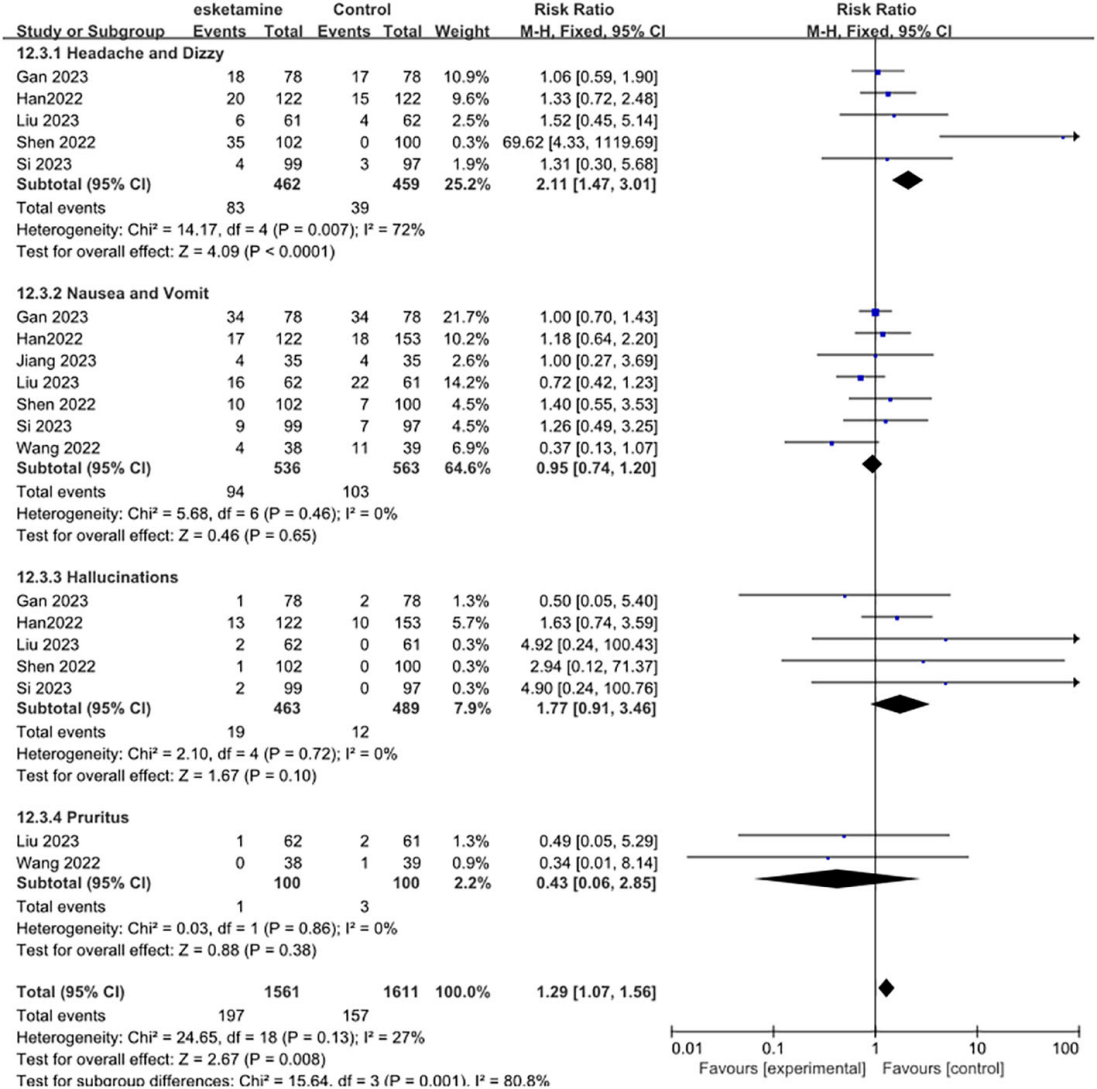
Meta-analysis of postoperative adverse effects. Including headache and dizzy, nausea and vomit, hallucinations, and pruritus.

Next, we examined the adverse effects of esketamine, including headache and dizziness, nausea and vomiting, hallucinations, and pruritus. Compared with control group, the use of esketamine had a higher risk of side effects. Significantly increased the risk of headaches and dizziness (RR 2.11, 95% CI [1.47, 3.01], P = 0.007, I^2^ = 72%). However, Nausea and vomit (RR 0.95, 95% CI [0.74, 1.20], P = 0.46, I^2^ = 0%), Hallucinations (RR 1.77, 95% CI [0.91, 3.46], P =0.72, I^2^ = 0%), Pruritus (RR 0.43, 95% CI [0.06, 2.85], P =0.86, I^2^ = 0%) was not statistically significant (**Figure 5**).

## 4 Discussion

In this meta-analysis, we pooled current studies on the efficacy of esketamine on postoperative depression to look for evidence of its effectiveness and safety. Based on the included literature, we found that the use of esketamine could prevent or reduce the level of postoperative depression. In the short term (1 week) and more than 1 month, it can effectively reduce the postoperative depression score and the incidence of postoperative depression. Intraoperative intravenous administration of esketamine and post-operative addition of PCIA showed that esketamine significantly alleviated postpartum depression symptoms in cesarean section patients. Meanwhile, through studies (Han et al. (20), Wang et al. (21), Yang et al. (23), Liu et al. (26)), it was found that the antidepressant effect of Esketamine was more obvious in the short-term postoperative period.

Almost all the reviewed studies have demonstrated the antidepressant effect of esketamine. Meanwhile, we found that the higher the dose of esketamine, the stronger its antidepressant effect [Wang et al. (21), Yang et al. (23)], the dose-effect relationship that exists within a certain range. However, Compared with the control group, patients receiving esketamine treatment had a higher risk of adverse reactions such as headache, dizziness. The dose range of esketamine for patients included in the meta-analysis is 0.1 to 2 mg/kg, with the most commonly used dose being 0.2 mg/kg. It is generally believed that as the dosage increases, adverse reactions relatively increase. However, there was no significant difference between the high dose group (1mg/kg) and the low dose group (<0.4mg/kg) in this meta-analysis. It may be that differences in dosage and administration (PCIA, Single dose, Continuous infusion) lead to inconsistent results.

It is possible that the effects of intraoperative medication and surgical methods contribute to the lack of evidence, it may also be because intraoperative intravenous antiemetic drugs such as ondansetron reduced the occurrence of postoperative nausea and vomiting, which remains to be studied further. Therefore, the optimal dose of esketamine for the treatment of postoperative depression is not known. Further clinical studies are needed to determine the appropriate dose to treat postoperative depression to reduce the risk of adverse reactions.

Studies have shown that esketamine and its subtypes are effective interventions for reducing depression in depressed patients and have powerful and beneficial effects in the short term. Robustness to various symptoms and relatively sustained antidepressant effects are also their advantages (28, 29). But for different surgical methods, the efficacy of esketamine in preventing depression still needs to be observed through clinical trials. [Shen et al, (25)] found no significant difference in antidepressant treatment with esketamine during surgery, which may be due to a shorter observation period compared to patients in other studies, or due to the patients in this study were relatively healthy or had no symptoms of depression before surgery. In addition, differences in drug dosage and administration time may also lead to differences in results. At the same time, the different depression assessment scales used in the included studies increased heterogeneity. Second, differences in follow-up time may affect the antidepressant effects of esketamine. Therefore, it is possible to reduce the adverse effects of esketamine by adjusting the dosing schedule and combining with other general anesthesia drugs in a reasonable study design. At the same time, further clinical studies are needed to investigate the efficacy and duration of esketamine in the treatment of perioperative depression.

In previous clinical studies, the NMDA receptor antagonist ketamine has demonstrated antidepressant efficacy. And esketamine is a chiral cyclic hexone with strong analgesic effect, which is the dexter of ketamine, which has a separate anesthetic effect like ketamine. As a new generation of isomeric drugs, esketamine acts in a mechanism similar to racemic ketamine, but it binds more to NMDA receptor and μ receptor and is about twice higher than that of racemic ketamine. However, it has shorter recovery time than ketamine and less central nervous system side effects than ketamine (30). Sarasso P, et al. select two case studies, suggest that esketamine-induced disembodiment allows for a transient window of psychological plasticity and enhanced sensitivity, where the body recovers its permeability to affective affordances (31). In addition, the parasympathetic effect of esketamine does not inhibit the hemodynamics of patients, and it also has advantages in the prevention of postoperative delusion. Therefore, Esketamine has the following advantages: low incidence of side effects such as hallucinations and rapid postoperative recovery.

Now esketamine has received regulatory approval for use in the treatment of refractory depression in the United States and elsewhere. It was approved by The US Food and Drug Administration (FDA) in 2019 for treatment-resistant depression. Meanwhile, there is increasing evidence to evaluate the efficacy and safety of esketamine as a treatment for depression. The FDA trial did not show any sustained or significant reduction in cognitive processes, and some studies suggest that improvements in cognitive areas may be related to improvements in depressive symptoms. A recent Delphi Panel showed a widespread heterogeneity in the management of TRD patients, study revealed a high level of consensus and agreement was obtained about the identification of esketamine nasal spray as the best option to antidepressants, emphasizing its integration into routine care within outpatient settings (32). At the same time, brief increases in blood pressure (33, 34) and heart rate (35) should not be ignored during the use of esketamine. Headache and nausea are also common, but often the symptoms are mild, transient, and easily controlled (36). These need to be timely detected and actively handled by the anesthesiologist and the nursing team during the perioperative and postoperative care period.

This analysis has some limitations. The disadvantage of this meta-analysis is that the surgical methods included in the study are not comprehensive, resulting in a relatively small sample size. Only a few surgical methods cannot reflect the comprehensive efficacy of esketamine on the prevention and improvement of postoperative depression, so the accuracy of the conclusions still needs to be verified. Furthermore, underrepresentation of non-obstetric surgeries, the lack of diversity in surgical procedures represented in the included studies, lack of standardized preoperative depression screening and the variability in depressive symptom measurement tools used across studies, as this could impact generalizability. Meanwhile, in the included studies, different analyses were conducted on anesthesia methods, administration methods, and doses based on the research results. Secondly, the level of preoperative depression and postoperative follow-up treatment lack standardized procedures, therefore, the duration and effect of esketamine cannot be evaluated. Furthermore, the heterogeneity in the selection of postoperative depression assessment tools across these studies has introduced variability in directly comparing the efficacy of esketamine among different trials. Therefore, randomized controlled trials with a larger sample size and similar scales are needed to confirm whether perioperative use of esketamine can help reduce postoperative depression symptoms.

## 5 Conclusion

The current meta-analysis shows that the perioperative application of esketamine is effective in reducing postoperative depression scores and pain intensity, particularly in obstetric and oncologic surgeries. However, esketamine increased the risk of nausea and vomiting, headache, hallucinations, and dizziness compared with placebo. Therefore, assess the differences in the effects of drug administration at various time points before, during, and after surgery, studying the optimal dosage and administration time of esketamine for postoperative antidepressant effects, while considering how to reduce the occurrence of adverse reactions in patients is still an exploratory issue in future clinical practice. To conduct a multicenter, randomized controlled trial to explore the antidepressant effects of different doses of esketamine in the perioperative period, and to define the minimum effective dose and the maximum tolerated dose. Administer prophylactic medications concurrently during the perioperative period to mitigate the risk of adverse reactions.

## Data Availability

The original contributions presented in the study are included in the article/supplementary material. Further inquiries can be directed to the corresponding authors.
